# Yeast as a simple eukaryotic model to study human diseases linked to membrane trafficking

**DOI:** 10.1186/2046-2530-4-S1-P31

**Published:** 2015-07-13

**Authors:** J De Craene, S Bär, J Laporte, V Marion, H Dollfus, S Friant

**Affiliations:** 1 UMR7156 Trafic Intracellulaire et Signalisation Lipidique, Universite de Strasbourg, Strasbourg, France; 2 Laboratoire de Physiopathologie des Maladies Neuromusculaires, IGBMC, Illkirch, France; 3 Unité INSERM U1112, Université de Strasbourg, Strasbourg, France

## 

Many human diseases are linked to mutations in genes encoding membrane trafficking proteins, among them the X-linked centronuclear myopathy (XLCNM) and ciliopathies. We use yeast *Saccharomyces cerevisiae *as a model system to study these human disease trafficking genes. It is a good model because yeast and human cells present similar intracellular organization and its membrane trafficking and metabolism are well characterized. We first studied the MTM1 gene, responsible for XLCNM, which codes for a myotubularin, a lipid phosphatase required for endosomal sorting. The heterologous expression of wild-type MTM1 or XLCNM patient mutants in yeast allowed comparing their *in vivo *phosphatase activity and their cellular function in trafficking. The yeast results showed that the phosphatase activity is not defective in patient mutants, further confirmed in the mouse *Mtm1 *KO model for myopathy [1].

Ciliopathies are complex genetic disorders that result from defects in the formation and/or function of the primary cilium. They include disorders such as Bardet-Biedl syndrome (BBS), Joubert syndrome (JBTS) and Alström syndrome (ALS) [2]. Cilia are involved in development and in signaling cascades. Despite, the identification of a large number of genes involved in ciliopathies (http://www.syscilia.org/goldstandard.shtml), the molecular and cellular mechanisms by which they cause the disease are largely unknown. In collaboration with the laboratory of Hélène Dollfus, we aim at understanding the cellular roles of membrane trafficking genes involved in ciliopathies by using the yeast model system. Indeed, organization of the primary cilium depends on membrane trafficking since many genes linked to ciliopathies are involved in membrane trafficking [3].
